# The influence of circulation weather types on the exposure of the biosphere to atmospheric electric fields

**DOI:** 10.1007/s00484-020-01923-y

**Published:** 2020-04-30

**Authors:** K. Kourtidis, K. Szabóné André, A. Karagioras, I.-A. Nita, G. Sátori, J. Bór, N. Kastelis

**Affiliations:** 1 Department. of Environmental Engineering, Democritus University of Thrace, 67100 Xanthi, Greece; 2 Also at ATHENA Research and Innovation Center in Information, Communication and Knowledge Technologies, ISLP Xanthi Branch, 67100 Xanthi, Greece; 3 Research Centre for Astronomy and Earth Sciences, Geodetic and Geophysical Institute, Csatkai u. 6-8, Sopron, 9400 Hungary; 4 Department of Research and Meteo Infrastructure Projects, Meteo Romania (National Meteorological Administration), Sos Bucuresti-Ploiesti 97, 013686 Bucharest, Romania; 5 Faculty of Geograghy and Geology, Alexandru Ioan Cuza University, Iasi, Bld. Carol I, nr. 65, Iasi, Romania; 6 Now at Environment, Maritime & Resilience Department, Jacobs, Cottons Centre, London, SE1 2QG UK

**Keywords:** Atmospheric electric field, Potential gradient, Schumann resonances, Circulation weather types

## Abstract

We present an analysis of the impact of circulation weather types (CT) on a factor that might influence biological systems and the human condition, the electric state of the atmosphere. We present results on the influence of CT to the magnitude, the direction (positive or negative), the fluctuation magnitude, and the short-term peaks of the atmospheric electric field (potential gradient, PG), using data from a station in Greece. CTs with high vorticity centers over Greece are associated with high positive and negative excursions of the PG, higher PG variability, and rain events. CTs with thinner 850–500 hPa layer are associated with higher daily mean values of fair-weather PG. We also examine the influence of CT on the frequency and amplitude of the naturally occurring extremely low-frequency electric field fluctuations known as Schumann resonances (SR) using data from a station in Hungary. The first and second mode SR frequencies are increased during CTs associated with higher 500 hPa geopotential heights and higher 850–500 hPa layer thickness. This hints to a lower-upper atmosphere coupling. So, CTs not only influence the general temperature and humidity conditions to which the biosphere is exposed, but they also affect its exposure to atmospheric electric fields.

## Introduction

Impacts of atmospheric parameters on human health have been documented for a long time and range from impacts on thermal comfort, vector-borne disease (e.g., McGregor 2011), children health (e.g., Vanos 2015), and fall and hip fractures. Factors that influence human health are diverse and range from temperature, changes in atmospheric pressure, humidity (e.g., Davis et al. 1996), and a variety of other factors. Reactions of the human organism are not the results of one or the other meteorological factor alone but the result of the interplay of different factors. It has been shown also that abrupt changes in weather have a large biometeorological impact.

Research on relationships between large-scale atmospheric circulation and different climatic and environmental variables has seen a revival in recent years, due to its usefulness in a variety of applications (e.g., Jacobeit 2010; Ramos et al. 2015 and references therein), including biometeorology (e.g., Kyselý and Huth 2010; Greene and Kalkstein 1996; Kassomenos et al. 2007) and electrification of the atmosphere as demonstrated by lightning (Pineda et al. 2010; Ramos et al. 2011).

Relationships between circulation weather types and human well-being have been studied by a number of authors (e.g., Lecha Estela 1998; Kassomenos et al. 2001; de Pablo et al. 2009).

The vertically oriented direct current (DC) voltage in the atmosphere, known as atmospheric electric field or potential gradient (PG), is almost certain to be influenced by the synoptic weather conditions, but no detailed analysis of this influence has been carried so far. PG during fair-weather conditions is around + 100 V/m, but during passage of electrified clouds, PG can increase to several hundreds of +/– V/m, and during discharges PG at ground level can increase to several ± 10,000 V/m. A resonance frequency of the electric field is also present in the atmosphere and is known as Schumann resonances (SR). The SR are naturally occurring fluctuations in the atmosphere (and most probably the oceans) that are globally present and have their main frequencies around 7.8 (first mode), 14 (second mode), and 20 Hz (third mode). For an introduction to SR, see Price (2016).

With the present paper, we aim to present a first analysis of the impact of CT not only on a variety of atmospheric variables that are known to influence human well-being but also on an additional factor, namely, the electric state of the atmosphere. According to some studies, the electric state of the atmosphere might influence biological systems (e.g., Perry 1900; Schauer et al. 2014; Morley and Robert 2018; Hunting et al. 2019) and also the human condition (König 1974; Cherry 2002; Mitsutake et al. 2005; Chevalier et al. 2012; Elhalel et al. 2019; Fdez-Arroyabe et al. 2020a, 2020b). As most studies of biometeorological influences to human beings are focused on meteorological variables, and as these might not only exhibit co-variation among them but might also co-vary with the electrical state of the atmosphere, we examine here the influence of the synoptic weather condition on meteorological variables and the electric state of the atmosphere with regard to the PG magnitude, its fluctuation, its excursion above certain thresholds, its polarity reversal, lightning occurrence, and SR frequency and amplitude.

The in-depth study of climate and its influence on biological systems requires an analysis of various weather situations or types. The basics of complex climatology were laid down by Fedorov and Chubukov (Fedorov and Chubukov 1963; Kozłowska-Szczęsna 1965). It is widely agreed that an approach involving analysis of individual meteorological elements fails to offer a full picture of the features of the climate because it lacks the crucial component of the mutual relationships between the meteorological elements. It is their combined effect which determines the type of climate. In this context, we introduce here the electric state of the atmosphere as an additional crucial bioclimatic element of weather types.

## Experiment and methods

The measurements of potential gradient, temperature, relative humidity, pressure, precipitation, and wind span for 7 years (2011–2017). Because the PG station was installed in early 2011, due to technical problems, we use only the data from March 11, 2011 onward until December 31, 2017.

The site is located on the Campus of Democritus University of Thrace, at a site (41.148^0^ N, 24.92°E, 75 m above sea level) near the town of Xanthi (population, 65,000), Greece. Briefly, the site is rural; it is situated 2.5 times the city radius from the center of nearby Xanthi. The site is not influenced by the city with regard to temperature due to distance and a very weak urban heat island effect (unpublished data). Only rarely the site might be influenced by city emissions (e.g., of aerosols), since the dominant wind direction (NNE during nighttime and S during daytime) is not from the city (the city lying E-SE (city center at ESE) of the site). More details about the site can be found at Kastelis and Kourtidis (2016).

PG is measured with a CS110, Campbell Scientific Co. Electric Field Meter (EFM), on a 2-m mast. Standard meteorological parameters (wind speed/direction, temperature, relative humidity, pressure, precipitation) were measured co-located with the EFM. Wind speed and direction were measured with a Wind Sentry Set (Model 03002 L, Young Co., U.S.A) and a wind vane with accuracy ± 0.5 m s^–1^ and ± 5o, respectively. Temperature and relative humidity were measured with a thermometer/hygrometer of ± 0.3 K and ± 1.5% accuracy (Model HygroClip S3, Rotronic Co., Switzerland). Pressure was measured with a barometric pressure sensor of 0.3 hPa accuracy (Model PTB110, Vaisala Co., Finland). Precipitation was measured with a tipping bucket rain gauge (Model 52,202, Young Co., U.S.A.). Data were recorded as 1-min means. A 1-min data were subsequently used to calculate 1-h data, which were used to calculate daily means which in turn were used to calculate monthly means. The PC used for data acquisition was synchronized in regular intervals to the NIST time server. More details about the instrumentation can be found at Kastelis and Kourtidis (2016). The fair-weather (FW) days were selected out of the dataset using both the FW classical definition and the 1965 one of the International Commission on Atmospheric Electricity (Israelsson 1978). FW PG was calculated as follows: First, we used only mean hourly values that were between 0 and 350 V/m. Secondly, we excluded from these hourly values those that had a standard deviation of their 1-min values > 100 V/m. These hours were classified as FW hours. The mean daily FW was calculated from FW hours, if there were > 12 FW hours for this day. We did not use the more up-to-date FW criteria by Harrison and Nicoll (2018) for two reasons: Firstly, they are most relevant for retrieving global circuit information from PG. Secondly, they require “absence of hydrometeors, aerosol and haze, as apparent through the visual range exceeding 2 km, negligible cumuliform cloud and no extensive stratus cloud with cloud base below 1500 m, and surface wind speed between 1 m s^−1^ and 8 m s^−1^”, and as we do not have cloud and cloud base info, we used another approach. We note that our approach conserves the requirement for the absence of hydrometeors through the requirement of standard deviation of 1-min values being less than 100 V/m.

Lightning data for all Europe from the BlitzOrtung lightning detection network (www.blitzortung.org) for the period January 1, 2015 to December 31, 2017 have been used to characterize CTs in Europe from the point of view of the distribution of lightning activity. The reason for not using the same period as for the PG measurements is that from 2015 onward due to the addition of sensors, the BlitzOrtung system has better locating accuracy. BlitzOrtung is based on magnetic and electric field detectors operating predominantly in the very low-frequency band (1–50 kHz). Sensors of the network are operated by volunteering partners worldwide. The density of the sensors in the network has been growing in recent years, and they are the highest in Europe. In this study, lightning data from BlitzOrtung were considered only from 2015 onward when the network covered the continent already well so that the obtained information represents lightning activity in the region correctly in the statistical sense. A study on the performance of the network in Japan (Narita et al. 2018) pointed out that BlitzOrtung reports less strokes (12–24%) than a professional local lightning detection network which operates also in the VLF band. The percentage of strokes detected by both BlitzOrtung and local lightning location networks was found to depend on the density of BlitzOrtung sensors in the area and varied between 5 and 26% in the examined cases. This may be due to the possibly not ideal installation environment for a number of BlitzOrtung sensors and can be related also to the strict detection requirements used by the BlitzOrtung network in order for the number of false detections to be reduced. The density of lightning strokes obtained from BlitzOrtung, therefore, must be interpreted with care. It must be taken into account that more strokes are detected in Central Europe where the density of the stations is higher. In order to examine the distribution of lightning strokes in each CTs, BlitzOrtung lightning stroke locations have been counted in cells determined by the ERA-Interim reanalysis database 0.75° × 0.75° grid over the area of interest (30° - 56°N, 4.5° - 45°E) on each day between 01/01/2015 and 31/12/2017. Then, the number of lightning strokes was averaged for all days corresponding to the same CT. Values corresponding to the grid cell of Xanthi (coordinates of the center of the grid cell #41.25°N 24.75°E) were considered as local lightning.

The circulation weather types were computed for Europe for each day. We use here a PXE (Pca-eXtreme scores reassigned by Euclidean distance) principal component analysis (PCA) categorization of 10 CT classes. PXE circulation weather types (from now on CT) were computed for the period of the measurements, i.e., 2011–2017.

The PXE method is based on principal component analysis where the initial centroids are obtained by using orthogonally rotated scores (through varimax rotation) that are calculated by the PCA in s-mode (Esteban et al. 2006). The PCs obtained are representing loading patterns in respect with the original data, and the final number of centroids (circulation types) can be selected by the user. However, this method is using the extreme scores criterion to calculate the circulation types, where for each PC and each phase (positive or negative), a circulation type is created by selecting only cases with absolute high scores (above 2 or below − 2) as well selecting cases with absolute low values (between − 1 and + 1). In this manner, each case can be represented by a PC. Since the number of the potential classes will be twice of the original number of PCs retained due to the positive and negative phases, the scores that are not having at least one case assigned to it will be removed (according to the extreme scores criteria). The final centroids are calculated as the mean score values of the cases assigned to them by Euclidean distance (Philipp et al. 2010).

Here, the PXE was created by using cost733 class software (Philipp et al. 2014) and reanalysis data from ERA-Interim (Dee et al. 2011). Daily mean sea-level pressure (SLP), the 500 hPa geopotential height (z500), the thickness (k) of the layer between the 850 hPa and 500 hPa isobaric surfaces, and the relative vorticity at 500 hPa were used in the input and classified. The number of centroids selected was 10. The static condition of the atmosphere is described by the height of a certain isobaric surface, e.g., the 500 hPa one. Differential variables, such as, e.g., the 500–850 hPa layer thickness, do not contribute to this (e.g., Bucher 1993).

There is a large number of weather and circulation type classification schemes (more than 70, see, e.g., Philipp et al. 2010), yielding for Europe anything between 9 and 10 and 40 CTs and using either only mean sea level pressure as input or SLP combined with one or some of the following: geopotential height, zonal and meridional wind components, precipitable water, and pressure at some reference heights. We opted for using the PXE classification since it offers a limited number of CTs, as this would facilitate the discussion at this stage, and also uses apart from Mean SLP alone also variables that give indications about the stability and the convection state of the atmosphere at some height.

Since no Schumann resonance (SR) measurements are available from Xanthi, but as we wanted to show that SR is also influenced by CT, we used the vertical electric field component of SRs recorded in the Széchenyi István Geophysical Observatory (47.63 N, 16.72E) near Nagycenk, Hungary (Sátori et al. 1996). Hourly data from 12:00 GMT are used. SR frequencies are a global manifestation and are mainly defined by the circumference of the Earth and the properties of lower ionosphere. While the resonant frequencies are determined solely by the geometry of the Earth-ionosphere cavity, peak frequencies of the resonance spectrum depend also on the distance between the excitation lightning source and the observer in the lossy Earth-ionosphere cavity (Nickolaenko and Hayakawa 2002). In this study, peak frequencies and amplitudes of the SR spectrum are analyzed as they have been obtained from the time series recorded at NCK using the method of complex demodulation (Sátori et al. 1996). The reader is advised to note that as Xanthi and Sopron may not lie within the same regime of the wide-area CT, the response in Xanthi may be somewhat different. On the other hand, the distance between the two sites is less than 1 Mm, their time difference is only around 1 h (thus not influenced by different summer/winter and day/night effects or local variations in the ionospheric height (Sentman and Fraser 1991)), and we use hourly data from 12:00 GMT, which should exclude also any ionospheric sunset/sunrise effects (Pechony et al. 2007; Sátori et al. 2007). Hence, it cannot be excluded that SR parameters at Nagycenk should not differ substantially from those at Xanthi.

## Results and discussion

### Circulation weather types

**Fig. 1:**
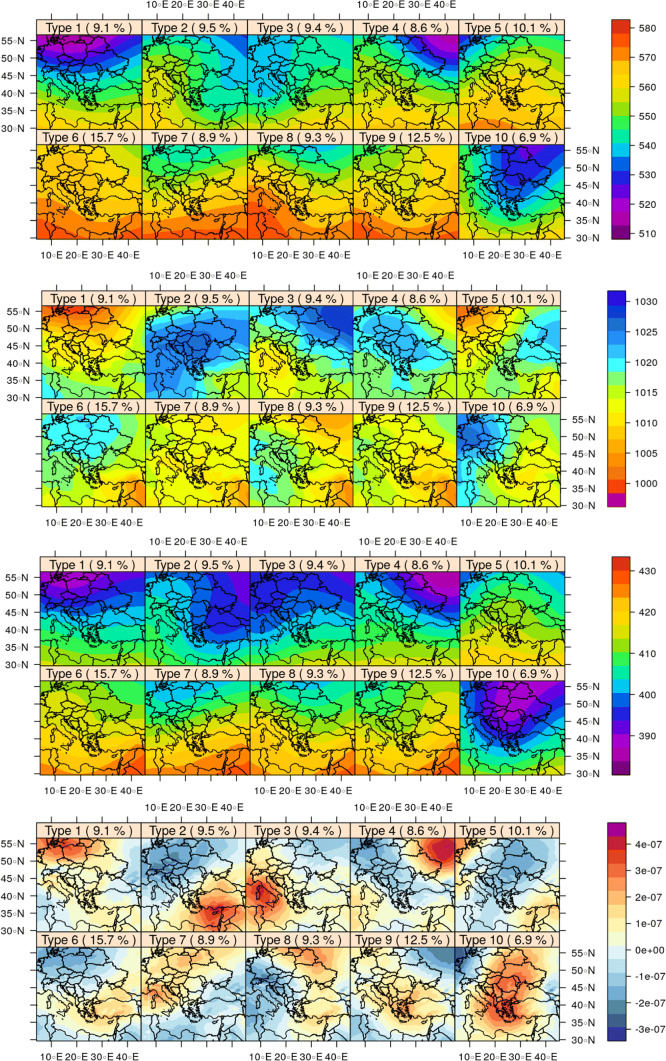
PXE circulation weather type with regard to (from top to bottom). Spatial patterns of geopotential at 500 hPa (10 m), sea level pressure (hPa), thickness between the 850 and 500 hPa isobaric surfaces (10 m), and relative vorticity at 500 hPa (s^−1^)

**Fig. 2:**
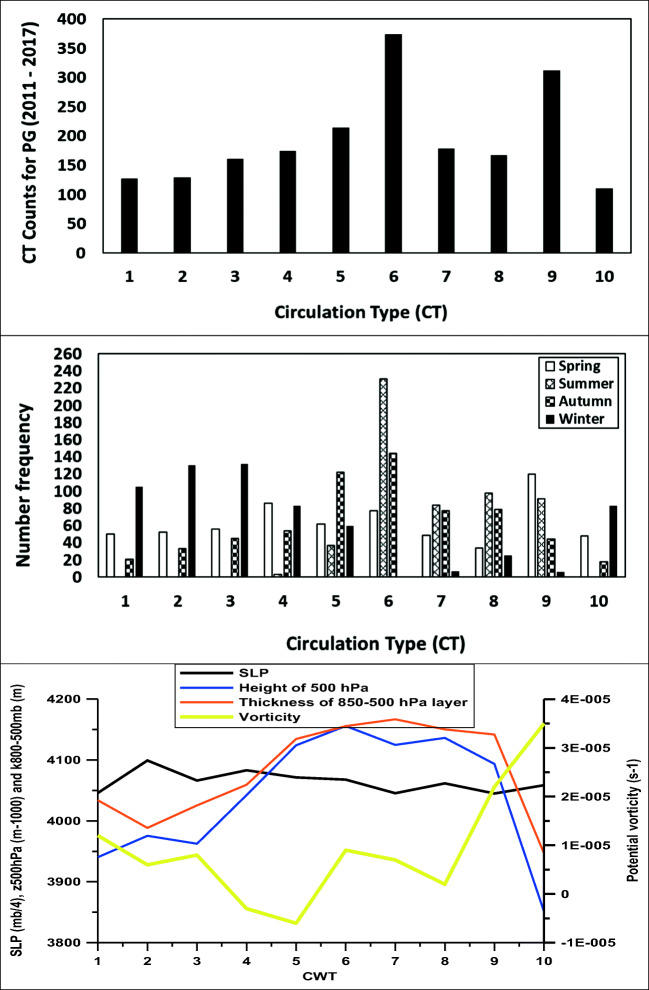
Number frequency of weather types (top only for days where PG data are available), number frequency of weather types per season (middle), and mean values over Xanthi of the parameters per CT involved in the PXE CT computation (bottom)

The synoptic meteorological analysis resulted in the categorization of each day to one of 10 PXE circulation weather types over Europe and the Eastern Mediterranean with regard to patterns of sea-level pressure (SLP), relative vorticity at 500 hPa, geopotential height at 500 hPa, and thickness of the layer between the 850 and 500 hPa isobaric surfaces, which are shown in Fig. 1 and described below. The seasonal frequency of these CTs and the mean values of the parameters involved in the PXE CT computation are shown in Fig. 2. Most CTs appear about 10% of the time each, except for CT10 which appears 6.9% of the time and CT9 and CT6 which appear for 12.5% and 15.7% of the time, respectively (see also Fig. 2).

#### CT1

This type is associated with a deep low over N Europe and results in W-SW surface flow over the site. This type of weather is encountered predominantly during winter but also during spring. Lightning activity over continental Europe, the Balkans, and Eastern Mediterranean is low (Fig. 7).

#### CT2

This type is associated with a high over the Balkans and results in NW flow over the site. Its seasonal frequency is the same as for CT1. Lightning activity over continental Europe, the Balkans, and Eastern Mediterranean is low (Fig. 7).

#### CT3

This type is associated with a high over Russia and a low over Italy, resulting in S surface flow over the site. This type of weather is encountered predominantly during winter but also during autumn and spring. The surface flow is such that it can result in Saharan dust transported to the site. It is known that intense Saharan dust outbreaks over Greece are associated with a low located in Central Europe with the south periphery of the low at Libya (e.g., Michaelides et al. 1999; Kaskaoutis et al. 2008). This is associated with an extended trough at 500 hPa geopotential height centered in Italy, which results in southwestern circulation over Greece (Kaskaoutis et al. 2008). This is the situation with CT3 (Fig. 1a, c). Additionally, CT3 is associated with high AOD over the Xanthi site as evident by mean MODIS AOD per CT 2011–2017 (figure not shown).

#### CT4

This type is associated with a relatively shallow low over most of continental Europe, resulting in W-WSW surface flow over the site. Compared with CT1, the center of high vorticity and the centers of shallow 500 hPa isobaric heights and shallow thickness of the 850 and 500 hPa isobaric surfaces are moved to the East, from N Europe to Russia. This type of weather is encountered predominantly during winter and spring but also during autumn.

#### CT5

This type is associated with lows over the Middle East and N Atlantic and high over S Russia and is encountered predominantly during autumn but also during the other seasons. The height of the 500 hPa isobaric and the thickness of the 850 and 500 hPa isobaric surfaces decrease gradually from N Africa to N Europe. Vorticity has negative values over most of Europe.

#### CT6

This type is associated with a high over NE Europe and a low over middle East, resulting in NE surface flow over the site. This type of weather is encountered predominantly during summer, and the resulting synoptic flow in the Aegean basin is called Etesians (e.g., Aristotle (330 BC); Repapis et al. 1978, Tyrlis and Lelieveld 2013, Anagnostopoulou et al. 2014). This is the most frequent CT, occurring about 15% of the time. Lightning activity over continental Europe, the Balkans, and Eastern Mediterranean is high (Fig. 7).

#### CT7

This type of weather is encountered during summer and autumn but also during spring. The height of the 500 hPa isobaric and the thickness of the 850 and 500 hPa isobaric surfaces decrease gradually from N Africa to N Europe. There is a NW to SE high to low vorticity gradient, the vorticity changing sign over the Balkans. Lightning activity over continental Europe, the Balkans, and Eastern Mediterranean is high (Fig. 7).

#### CT8

This type of weather is encountered during summer and autumn but also during spring. The height of the 500 hPa isobaric and the thickness of the 850 and 500 hPa isobaric surfaces decrease gradually from N Africa to N Europe. Lightning activity over continental Europe, the Balkans, and Eastern Mediterranean is high (Fig. 7).

#### CT9

For 80% of the cases, this type of weather is encountered during spring and summer. The height of the 500 hPa isobaric and the thickness of the 850 and 500 hPa isobaric surfaces decrease gradually from N Africa to N Europe. Vorticity has high positive values over the Balkans, with the center of the high vorticity area located over Greece. Surface flow is eastern. Lightning activity over continental Europe, the Balkans, and Eastern Mediterranean is high (Fig. 7).

#### CT10

**Fig. 3:**
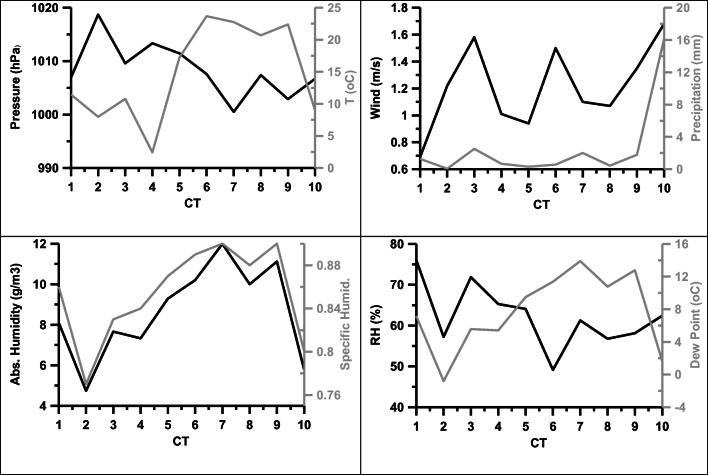
Mean daily pressure, temperature, wind speed, precipitation, absolute and specific humidity, relative humidity, and dew point per CT. The means were calculated from the daily mean values

This type is associated with a high over W Europe and a shallow low over Eastern Mediterranean, resulting in E-NE surface flow over the site. This type of weather is encountered about 7% of the time, predominantly during winter but also during spring. It is associated with high positive vorticity at 500 hPa, a low 500 hPa isobaric surface, and a thin 850–500 hPa thickness as well as steep gradients of the latter two from N to S. The high positive vorticity over Greece, the Balkans, and Eastern Mediterranean (Fig. 1) means that there will be considerable updraft during CT10. However, temperature and humidity (absolute and relative) are low at Xanthi (Fig. 3), and hence, thundercloud formation over the site will be limited.

The most frequent weather type over Europe and the Eastern Mediterranean is CT6. Weather types 1–4 and 10 occur predominantly in winter, CT5 in autumn and winter, while CTs 6–9 occur mostly in spring, summer, and autumn. The former are associated to lower absolute humidity (Fig. 3).

Statistics on the occurrence of the 10 CT classes are given in Fig. 2 which presents the mean values over Xanthi of the parameters per CT involved in the PXE CT computation. It is found that the height of the 500 hPa isobaric and the thickness of the 800–500 hPa layer are higher during CTs 6–9 (are mainly summer, autumn, and spring cases) and lower during winter cases. The vorticity seems to increase for CTs 8–10 when surface pressure is almost stable.

### Meteorological variables

The means of the daily values of meteorological parameters 2011–2017 during each CT are shown in Fig. 3. The mean temperature, dew point, and humidity, absolute and specific, of each CT correlate very well with the mean thickness of the 850–500 hPa layer over Xanthi for this CT (*r*^2^ = 0.68, 0.87, 0.86, and 0.83, respectively). The mean wind velocity exhibits some moderate correlation with mean vorticity over Xanthi at 500 hPa (*r*^2^ = 0.34). The correlation holds also for the time series of the respective variables, although with lower *r*^2^. Considerably more rain is associated with CT10 than with any other CT.

### Potential gradient

**Fig. 4:**
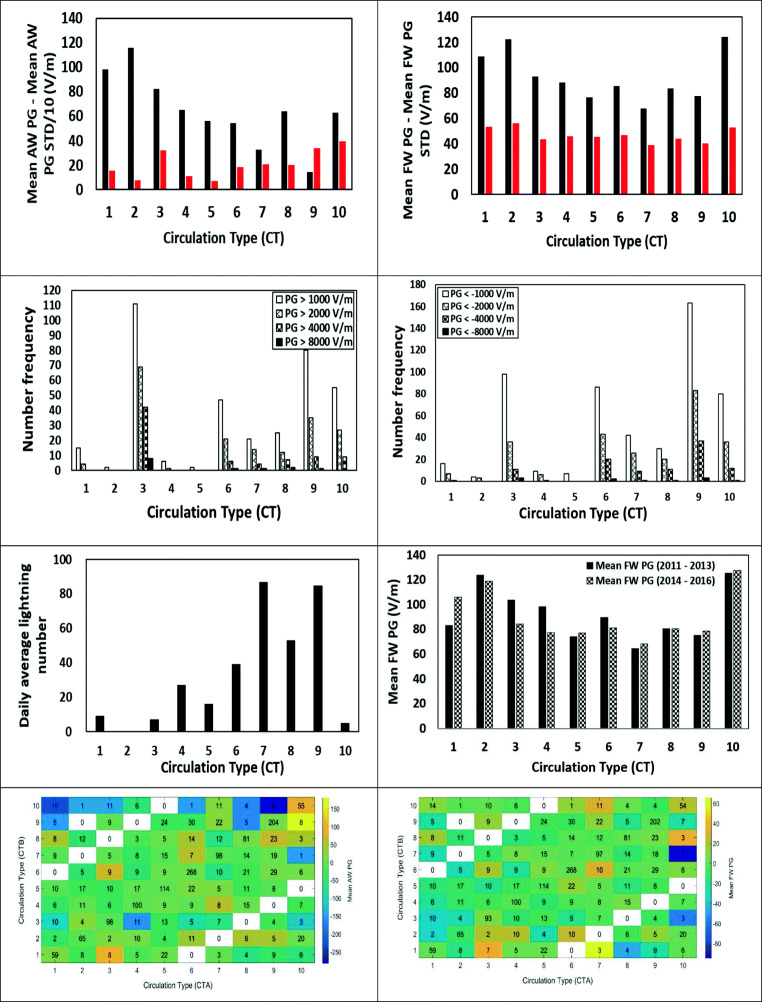
Mean daily AW PG 2011–2017 per CT over Europe and the corresponding mean standard deviation (red), the latter calculated from the hourly means for each day (upper left). Mean daily FW PG 2011–2017 per CT over Europe and the corresponding mean standard deviation (red); the latter calculated from the hourly means for each day (upper right). Number frequency of occurrences of mean hourly AW PG > 1000 V/m, PG > 2000 V/m, PG > 4000 V/m, and PG > 8000 (upper middle left) and number frequency of PXE weather types with occurrences of mean hourly AW PG < − 1000 V/m, PG < − 2000 V/m, PG < − 4000 V/m, and PG < − 8000 (upper middle right) per CT. Note the different vertical occurrence scales. Mean number of lightning discharges per day (lower middle left) per CT and mean FW PG per CT for two subperiods of 2011–2017 (lower middle right). Mean change in daily AW PG per change of CT from A to B (lower left) and mean change in daily FW PG per change of CT from A to B (lower right). The numbers in each cell denote the number of days a change from CT A to CT B occurred. The means were calculated from the daily mean values

The mean daily values per CT of all-weather (AW) PG 2011–2017 and the corresponding daily mean standard deviation, the latter calculated from the hourly mean AW PG values for each day, as well as the mean daily values of fair-weather (FW) PG 2011–2017, and the corresponding daily mean standard deviation calculated from the hourly mean FW PG values for each day, are shown in Fig. 4. We also split the values of hourly PG in bands of 8000, 4000, 2000, and 1000 V/m, both positive and negative, which are also shown in Fig. 4. If we split our measuring period in two, the observed changes in mean PG per CT still hold (Fig. 4).

The mean FW PG per CT anticorrelates strongly with the mean height of the 500 hPa isobaric (*r*^2^ = 0.74) per CT. These anticorrelations hold also for the respective time series, although with a much lower *r*^2^ (*r*^2^ = 0.13).

The highest positive excursions of hourly PG above + 1000 V/m and even + 8000 V/m occur for CT3 followed by CT9. CT10 also is associated with high positive/negative PG excursions (is a mainly winter CWT characterized by strong advection and E-NE flow in the surface). This possibly means transportation of particles from the neighboring countries. Finally, the most frequent regime, CWT 6, is associated with smaller PG excursions, mainly negative, indicating a healthier impact to the people. CT3 can result in Saharan dust transport over the site, and Saharan dust is known to be charged (Ulanowski et al. 2007; Nicoll et al. 2011; Yair et al. 2016). Yair et al. (2016) also observed that during a dust storm in the Negev desert, PG values fluctuated between + 1000 and + 8000 V/m. Given the human health significance of desert dust in many countries and the increased efficiency of deposition to the lungs of charged particles (e.g., Bailey 1997) as well as that surface charge is a key factor influencing lung inflammation (Kim et al. 2016), this observation bears also bioclimatological significance.

**Fig. 5:**
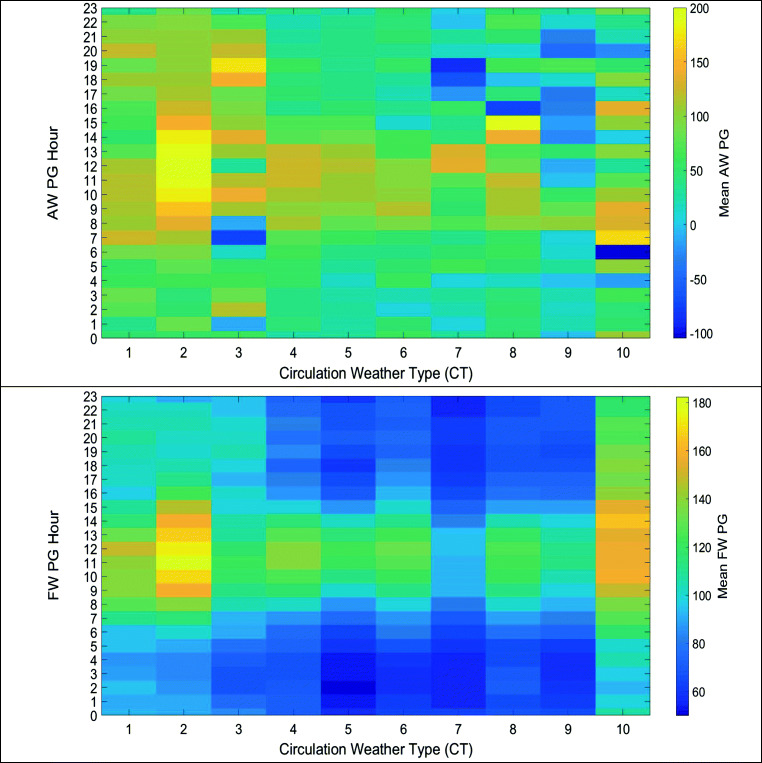
Mean hourly AWPG and FWPG per CT

As abrupt changes in weather parameters are known to influence health and well-being, we examined the changes in the mean daily values of all-weather and fair-weather PG when the CT changes from one type to another (Fig. 4, lower panels). As can be seen, the most frequent daily changes are from one weather type to the same (diagonal in the figures), and these are not associated with large changes in the PG. However, changes from one weather type to some other do occur, and some of these are associated with large changes in the PG (either in the positive or in the negative direction). Furthermore, CTs are found to also influence the daily pattern of PG (Fig. 5).

**Fig. 6:**
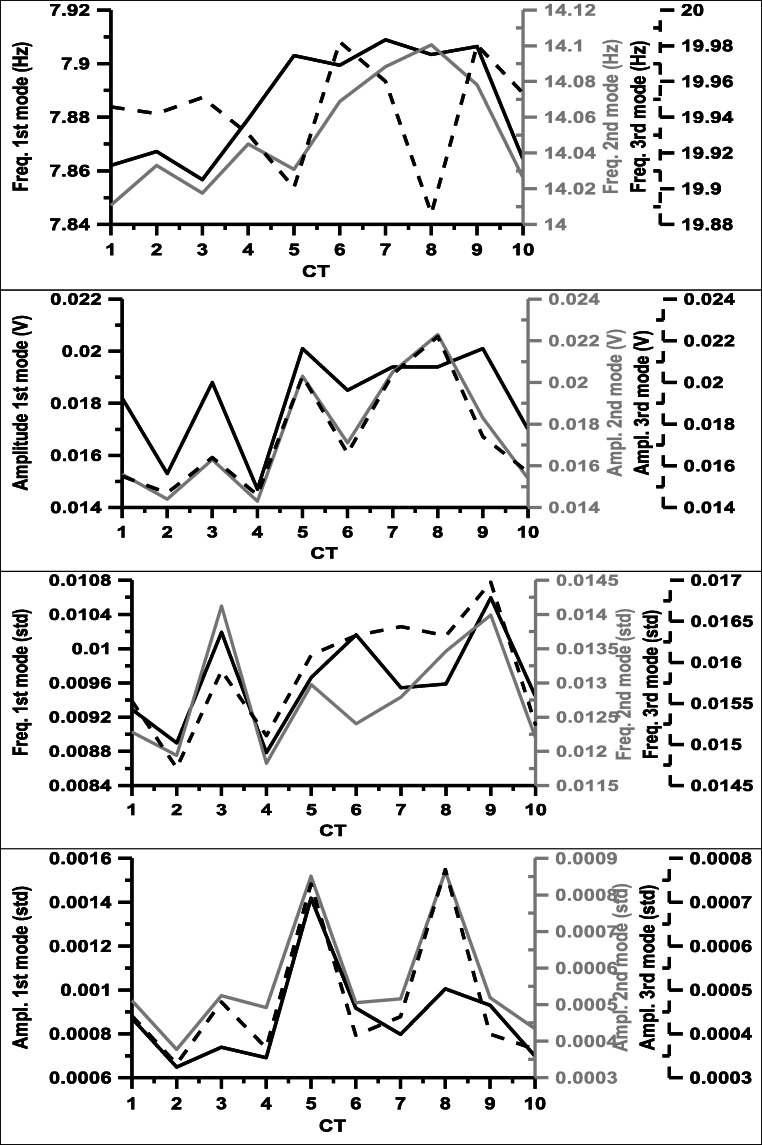
Mean frequencies and amplitudes of the first, second, and third mode Schumann resonances at 12:00 GMT per CT and mean standard deviation thereof of the hourly measurements at 12:00 GMT

**Fig. 7:**
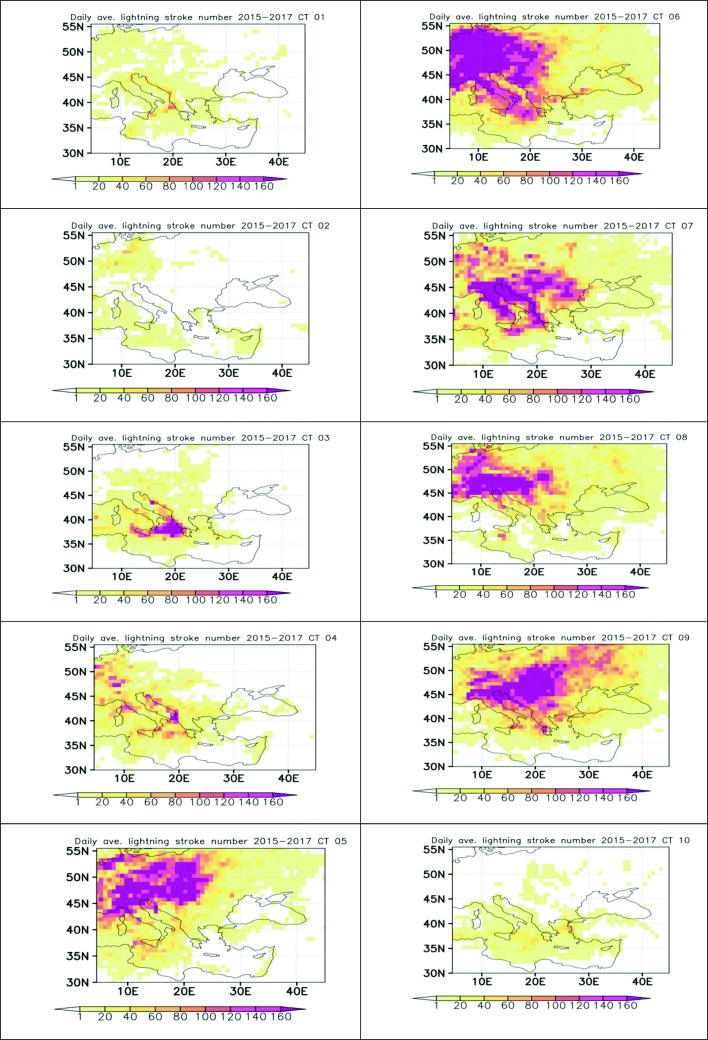
Daily average lightning density over Europe for the 10 CTs per ERA-Interim grid cell

### Schumann resonances

SR frequencies (Fig. 6) of the first and second modes are shifted to higher values in case of CTs 5–9 and 6–9, respectively. Regarding the third mode, frequencies of only the CT6 and CT9 are somewhat higher than those for the other CTs, while the frequencies are low with CT5 and CT8. SR amplitudes are higher for the 5, 7–9 CTs in case of the second and third mode, but not for the first mode. Note that CTs 2 and 4 have utterly small amplitudes in case of all 3 considered modes. SR frequencies of the first and second modes and amplitudes of all 3 modes tend to be higher for CTs with higher 500 hPa isobaric and thicker 500–850 hPa layer. CTs that produce higher first SR frequencies over Sopron have generally lower PG over Xanthi.

Comparing these tendencies with the distributions of meteorological parameters among CTs, the average temperature shows similar features. High frequencies in the first and second SR modes appear in CTs where the average temperature is relatively high. Additionally, relatively low temperature in CT4 matches with low values of the SR amplitudes in all three considered modes. Some similarity with the special humidity and dew point distributions can also be observed. These parameters have very low values in CT2 similarly to the SR amplitudes. The relation of SRs to temperature has been suggested by Williams (1992).

The interpretation of the amplitude and frequency shifts in connection with CTs in Europe is challenging, because the intensity and global distribution of thunderstorm activity as well as the location of NCK station in the structure of the nodal lines of the SR resonance modes need to be taken into account (Price 2016). The results above are nevertheless interesting since they hint to a lower-upper atmosphere coupling.

The variations in SR peak frequencies and intensity between different CTs is quite interesting for biometeorology, as SR appears to affect human condition (Cherry 2002; Mitsutake et al. 2005; Elhalel et al. 2019). Since various biometeorological studies have linked weather types to blood pressure and other manifestations of cardiac functioning (e.g. Morabito et al. 2008) and some authors have found possible coupling between SR and cardiac function (Mitsutake et al. 2005; Elhalel et al. 2019), in view of the results presented here, it appears that biometeorological studies may benefit from the combined use of meteorological and electric atmospheric variables.

### Lightning

Weather types with higher 500 hPa isobaric altitudes over Xanthi and low winter occurrence produce more lightning over the site.

Regarding lightning over the whole European domain, it appears (Figs. 7and 1) that weather types produce more lightning over regions with higher 500 hPa isobaric height and thicker 500–850 hPa layer.

## Conclusions

In this paper, an attempt was made to find possible connections with circulation weather types, potential gradient values, and other indicators of the electric state of the atmosphere.

The highest positive excursions of hourly PG above + 1000 V/m and even + 8000 V/m occur for circulation types associated with Saharan dust transport over the site. Saharan dust is known to be charged and is also of human health significance. Increased efficiency of deposition to the lungs of charged particles and surface charge being a key factor influencing lung inflammation, this observation is of high bioclimatological significance. We find also interesting correlations of the electrical state of the atmosphere with the NE-E summer Etesian winds that might also carry particles from the East.

It is found that circulation types affect the near-ground electric state of the atmosphere in terms of DC electric field magnitude, DC electric field fluctuations, DC electric field polarity, lightning, electric field frequency (SR), and amplitude. It is also found that certain changes from one CT to another cause large changes in the DC electric field magnitude.

Flemming (1996) stressed that “the admixtures and pollutants pertaining to air quality criteria belong to the atmosphere and therefore, of course, to the field of meteorology, and due to their possible effects also to human biometeorology”. We note that atmospheric electricity also belongs to the field of meteorology and, due to its possible effects, also to biometeorology. Because large variations in aspects of the atmospheric electric environment are observed with different circulation weather types, the atmospheric electric environment deserves to be included in CT-related bioclimatic studies.

It appears to us that with reasonable effort, it might soon be also possible to include certain aspects of the atmospheric electric field to biometeorological weather forecasts.

## References

[CR1] Anagnostopoulou C, Zanis P, Katragkou E, Tegoulias I, Tolika K (2014). Recent past and future patterns of the Etesian winds based on regional scale climate model simulations. Clim Dyn.

[CR2] Aristotle (330 BC) Meteorologica, Book B, Chapter 5, 361b, 362a, 365a (in Greek)

[CR3] Bailey AG (1997). The inhalation and deposition of charged particles within the human lung. J Electrost.

[CR4] Bucher K (1993) Ein Verfahren zur objektiven Klassifikation des Wetters unter biosynoptischen Gesichtspunkten. Berichte des Deutschen Wetterdienstes 183, 139 p., Deutcher Wetter Dienst, Offenbach

[CR5] Cherry N (2002). Schumann resonances, a plausible biophysical mechanism for the human health effects of solar/geomagnetic activity. Nat Hazards.

[CR6] Chevalier G, Sinatra ST, JOschman JL, Sokal K, Sokal P (2012). Review article - Earthing: health implications of reconnecting the human body to the Earth’s surface electrons. J Environ Public Health.

[CR7] Davis RE, McGregor GR, Enfield KB (1996). Humidity: a review and primer on atmospheric moisture and human health. Environ Res.

[CR8] de Pablo F, Tom’as C, Soriano LR, Diego L (2009). Winter circulation weather types and hospital admissions for cardiovascular, respiratory and digestive diseases in Salamanca, Spain. Int J Climatol.

[CR9] Dee DP, Uppala SM, Simmons AJ, Berrisford P, Poli P, Kobayashi S, Andrae U, Balmaseda MA, Balsamo G, Bauer P, Bechtold P, Beljaars ACM, van de Berg L, Bidlot J, Bormann N, Delsol C, Dragani R, Fuentes M, Geer AJ, Haimberger L, Healy SB, Hersbach H, Hólm EV, Isaksen L, Kållberg P, Köhler M, Matricardi M, McNally AP, Monge-Sanz BM, Morcrette J-J, Park B-K, Peubey C, de Rosnay P, Tavolato C, Thépaut J-N, Vitart F (2011). The ERA-Interim reanalysis: configuration and performance of the data assimilation system. Q J R Meteorol Soc.

[CR10] Elhalel G, Price C, Fixler D, Shainberg A (2019). Cardioprotection from stress conditions by weak magnetic fields in the Schumann resonance band. Nat Sci Rep.

[CR11] Esteban P, Martin-Vide J, Mases M (2006). Daily atmospheric circulation catalogue for Western Europe using multivariate techniques. Int J Climatol.

[CR12] Fdez-Arroyabe P, Fornieles-Callejón J, Santurtún A, Szangolies L, Donner RV (2020). Schumann resonance and cardiovascular hospital admission in the area of Granada, Spain: an event coincidence analysis approach. Sci Total Environ.

[CR13] Fdez-Arroyabe P, Salcines Suárez CL, Nita I-A, Kassomenos P, Petrou E, Santurtún A (2020). Electrical characterization of circulation weather types in northern Spain based on atmospheric nanoparticles measurements: a pilot study. Sci Total Environ.

[CR14] Fedorov EE, Chubukov LA, Chubukov LA (1963). Principles of complex climatology, its development and present state. Problems of Complex Climatology.

[CR15] Flemming G (1996). The importance of air quality in human biometeorology. Int J Biometeorol.

[CR16] Greene JS, Kalkstein LS (1996). Quantitative analysis of summer air masses in the eastern United States and an application to human mortality. Clim Res.

[CR17] Harrison RG, Nicoll K (2018). Fair weather criteria for atmospheric electricity field measurements. J Atmos Sol Terr Phys.

[CR18] Hunting ER, Harrison RG, Bruder A, van Bodegom PM, van der Geest HG, Kampfraath AA, Vorenhout M, Admiraal M, Cusell C, Gessner MO (2019) Atmospheric electricity influencing biogeochemical processes in soils and sediments. Front Physiol 10. 10.3389/fphys.2019.00378 10.3389/fphys.2019.00378 PMC6477044 31040789

[CR19] Jacobeit J (2010). Classifications in climate research. Phys Chem Earth.

[CR20] Kaskaoutis DG, Kambezidis HD, Nastos PT, Kosmopoulos G (2008). Study on an intense dust storm over Greece. Atmos Environ.

[CR21] Kassomenos P, Gryparis A, Samoli E, Katsouyanni K, Lykoudis S, Flocas HA (2001). Atmospheric circulation types and daily mortality in Athens, Greece. Environ Health Perspect.

[CR22] Kassomenos PA, Gryparis A, Katsouyanni K (2007). On the association between daily mortality and air mass types in Athens, Greece during winter and summer. Int J Biometeorol.

[CR23] Kastelis N, Kourtidis K (2016). Characteristics of the atmospheric electric field and correlation with CO_2_ at a rural site in southern Balkans. Earth Planets Space.

[CR24] Kim J, Chankeshwara SV, Thielbeer F, Jeong J, Donaldson K, Bradley M, Cho WS (2016). Surface charge determines the lung inflammogenicity: a study with polystyrene nanoparticles. Nanotoxicology.

[CR25] König HL, Persinger MA (1974). Behavioural changes in human subjects associated with ELF electric fields. ELF and VLF electromagnetic field effects.

[CR26] Kozłowska-Szczęsna T (1965). New soviet publications in the field of complex climatology. Int J Biometeorol.

[CR27] Kyselý J, Huth R (2010). Relationships between summer air masses and mortality in Seoul: comparison of weather-type classifications. Phys Chem Earth.

[CR28] Lecha Estela LB (1998). Biometeorological classification of daily weather types for the humid tropics. Int J Biometeorol.

[CR29] McGregor GR (2011). Human biometeorology. Prog Phys Geogr.

[CR30] Michaelides S, Evripidou P, Kallos G (1999). Monitoring and predicting Saharan Desert dust events in the eastern Mediterranean. Weather.

[CR31] Mitsutake G, Otsuka K, Hayakawa M, Sekiguchi M, Corndlissen G, Halberg F (2005). Does Schumann resonance affect our blood pressure?. Biomed Pharmacother.

[CR32] Morabito M, Crisci A, Orlandini S, Maracchi G, Gensini GF, Modesti PA (2008). A synoptic approach to weather conditions discloses a relationship with ambulatory blood pressure in hypertensives. Am J Hypertens.

[CR33] Morley EL, Robert D (2018). Electric fields elicit ballooning in spiders. Curr Biol.

[CR34] Narita T, Wanke E, Sato M, Sakanoi T, Kumada A, Kamogawa M, Hirohiko I, Harada S, Kameda T, Tsuchiya F, Kaneko E (2018) A study of lightning location system (blitz) based on VLF sferics. IEEE 34th International Conference on Lightning Protection, doi:10.1109/iclp.2018.8503311

[CR35] Nickolaenko AP, Hayakawa M (2002). Resonances in the Earth-ionosphere cavity.

[CR36] Nicoll KA, Harrison RG, Ulanowski Z (2011). Observations of Saharan dust layer electrification. Environ Res Lett.

[CR37] Pechony O, Price C, Nickolaenko AP (2007). Relative importance of the day-night asymmetry in Schumann resonance amplitude records. Radio Sci.

[CR38] Perry J (1900). Atmospheric electricity and disease. Nature.

[CR39] Philipp A, Bartholy J, Beck C, Erpicum M, Esteban P, Fettweis X, Huth R, James P, Jourdain S, Kreienkamp F, Krennert T, Lykoudis S, Michalides SC, Pianko-Kluczynska K, Post K, Rasilla Álvarez D, Schiemann R, Spekat A, Tymvios FS (2010). Cost733cat–a database of weather and circulation type classifications. Phys Chem Earth.

[CR40] Philipp A, Beck C, Huth R, Jacobeit J (2014). Development and comparison of circulation type classifications using the COST 733 dataset and software. Int J Climatol.

[CR41] Pineda N, Esteban P, Trapero L, Solera X, Beck C (2010). Circulation types related to lightning activity over Catalonia and the principality of Andorra. Phys Chem Earth, Parts A/B/C.

[CR42] Price C (2016). ELF electromagnetic waves from lightning: the Schumann resonances. Atmosphere.

[CR43] Ramos AM, Ramos R, Sousa P, Trigo RM, Janeira M, Prior V (2011). Cloud to ground lightning activity over Portugal and its association with circulation weather types. Atmos Res.

[CR44] Ramos AM, Barriopedro D, Dutra E (2015). Circulation weather types as a tool in atmospheric, climate, and environmental research. Front Environ Sci.

[CR45] Repapis C, Zerefos C, Tritakis B (1978). On the Etesians over the Aegean. Proc Acad Athens.

[CR46] Sátori G, Szendrői J, Verő J (1996). Monitoring Schumann resonances - I. Methodology. J Atmos Terr Phys.

[CR47] Sátori G, Neska M, Williams E, Szendrői J (2007). Signatures of the non-uniform earth-ionosphere cavity in high-time resolution Schumann resonance records. Radio Sci.

[CR48] Schauer R, Risgaard-Petersen N, Kjeldsen KU, Tataru Bjerg JJ, Jørgensen BB, Schramm A, Nielsen LP (2014). Succession of cable bacteria and electric currents in marine sediment. ISME J.

[CR49] Sentman DD, Fraser BJ (1991). Simultaneous observations of Schumann resonances in California and Australia: evidence for intensity modulation by the local height of the D region. J Geophys Res.

[CR50] Tyrlis E, Lelieveld J (2013). Climatology and dynamics of the summer Etesian winds over the eastern Mediterranean. J Atmos Sci.

[CR51] Ulanowski Z, Bailey J, Lucas PW, Hough JH, Hirst E (2007). Alignment of atmospheric mineral dust due to electric field. Atmos Chem Phys.

[CR52] Vanos JK (2015). Children’s health and vulnerability in outdoor microclimates: a comprehensive review. Environ Int.

[CR53] Williams ER (1992). The Schumann resonance: a global tropical thermometer. Science.

[CR54] Yair Y, Katz S, Yaniv R, Ziv B, Price C (2016). An electrified dust storm over the Negev desert, Israel. Atmos Res.

